# Supplementary material for: Chromogranin A and catestatin regulate pancreatic islet homeostasis, endocrine function, and neurotransmitter signaling
Source: Commun Biol. 2025 Nov 26;8:1684. doi: 10.1038/s42003-025-09135-z (PMC12658243; doi:10.1038/s42003-025-09135-z)
Supplement: Supplementary file 1 — Supplementary material [file 42003_2025_9135_MOESM1_ESM.pdf]

Supplementary Material

Chromogranin A and catestatin regulate pancreatic islet homeostasis, endocrine function, and neurotransmitter signaling

Elke M. Muntjewerff[1], Dali Epremidze[1], Mariya Nezhyva[2], Satadeepa Kal[3], Theresa V. Rohm[4], Kechun Tang[3], Kailash Singh[1], Daniel Espes[1,5,6], Suborno Jati[7], Marleen Bootsma1, Atef Mahmoud Mannaa[8,9], Hiromi Ikebuchi[1], Anna M. Nilsson[2,10], Mahadevan Rajasekaran[11], Per E. Andrén[2,10], Erik T. Jansson[2], Sushil K. Mahata[11,12]*, & Gustaf Christoffersson[1,6]*

[1]Department of Medical Cell Biology, Uppsala University, Uppsala, Sweden
[2]Department of Pharmaceutical Biosciences, Uppsala University, Uppsala, Sweden
[3]Veterans Medical Research Foundation, San Diego, CA, USA
[4]Division of Endocrinology and Metabolism, Department of Medicine, University of California San Diego, La Jolla, CA, USA
[5]Department of Medical Sciences, Uppsala University, Uppsala, Sweden
[6]Science for Life Laboratory, Uppsala University, Uppsala, Sweden
[7]Department of Chemistry and Biochemistry, University of California San Diego, La Jolla, CA, USA
[8]INSERM U1192, Laboratoire Protéomique, Réponse Inflammatoire & Spectrométrie de Masse (PRISM), Université de Lille, Lille, France
[9]Borg AlArab Higher Institute of Engineering and Technology, New Borg Al Arab City, Alexandria, Egypt
[10]Spatial Mass Spectrometry, Science for Life Laboratory, Uppsala University, Uppsala, Sweden
[11]VA San Diego Healthcare System, San Diego, CA, USA
[12]Department of Medicine, University of California San Diego, La Jolla, CA, USA

*** Correspondence:**
Gustaf Christoffersson
ORCID: 0000-0002-9640-9702

Department of Medical Cell Biology
Uppsala University
Husargatan 3
75123 Uppsala, Sweden
Email: gustaf.christoffersson@scilifelab.uu.se
Tel: +4618-4714325

Sushil K. Mahata, Ph.D.
ORCID: 0000-0002-8300-9873

Metabolic Physiology & Ultrastructural Biology Laboratory
Department of Medicine
University of California San Diego
9575 Gilman Drive
La Jolla, CA 92093-0732, USA
Email: smahata@health.ucsd.edu
Tel: +1 (858) 552-8585, extension 2637

Supplementary Tables: 2
Supplementary Figures: 14
Supplementary Method Figures: 4

Supplementary Table 1. Characteristics of healthy and T1D participants.

Diabetes onset (year)	Gender (M/F)	Age (year)	BMI (kg/m^2)	C-peptide (nmol/l)
1978	M	42	27	<0,01
1999	M	39	28,7	<0,01
1989	M	43	29,3	<0,01 *
1997	M	35	25,1	<0,01
1991	M	30	27,2	<0,01
2005	F	21	22,9	<0,01
1999	M	45	27,7	
1990	F	43	22,3	
1988	F	42	26,8	<0,01
1995	M	27	30,7	<0,01
2010	M	24	22,9	<0,01
1995	M	38	24,8	0,05 *
2001	M	49	26,3	<0,01
1997	M	35	25,6	0,04
2003	F	23	24,2	<0,01
1987	M	35	24,2	<0,01
1994	F	37	25,1	<0,01
1997	M	40	22,6	<0,01
2001	F	25	25,4	<0,01
2002	F	25	23,3	<0,01
1999	M	45	27,3	0,4
1994	F	41	30,5	0,05
1998	M	33	35,5	<0,01
1981	M	41	24,8	0,01
Healthy	F	51	27,1	0,5
Healthy	F	26	22,5	0,5
Healthy	F	26	20,7	0,5
Healthy	F	36	22,1	0,5
Healthy	M	39	22,8	0,4
Healthy	F	23	21,1	0,57
Healthy	F	25		
Healthy	F	25		
Healthy	M	26		
Healthy	M	30		
Healthy	M	23		
Healthy	M	58	23,9	0,7
Healthy	M	26	21,7	0,4

Characteristics of individuals from whom blood samples were used in the CST ELISA in main Fig. 1. The table displays year of diabetes onset (or healthy), gender: male (M) or female (F), age in years, body mass index (BMI) expressed in units of kilograms (kg) divided by square of height in metres (m^2) and C-peptide levels in nanomoles (nmol) per litre (l).

Supplementary Table 2. Metabolites identified and validated in the pancreas.

**Analyte name**	**Analyte short name**	**Theoretical m/z**
Serotonin	5HT TT	444.2062 m/z ± 7.7 ppm
Gamma-Aminobutyric acid	GABA_H2O TT	353.1642 m/z ± 8.1 ppm
Histamine	Histamine TT	379.191 m/z ± 9.4 ppm
Cysteine	Cysteine TT	389.1321 m/z ± 9 ppm
Taurine	Taurine TT	393.1267 m/z ± 8 ppm
Creatine	Creatine TT	399.1807 m/z ± 12 ppm
Spermidine	Spermidine TT	413.2694 m/z ± 7.2 ppm
Histidine	Histidine TT	423.1806 m/z ± 7.4 ppm
L-tyrosine	Tyrosine TT	449.1851 m/z ± 10 ppm
Spermine	Spermine TT	470.327 m/z ± 10 ppm
Hypoxantine	Hypoxantine TT	404.1503 m/z ± 9 ppm

The table lists analytes and their short name used in this study. Theoretical mass of the analyte is shown in (m) divide by charge number (z).

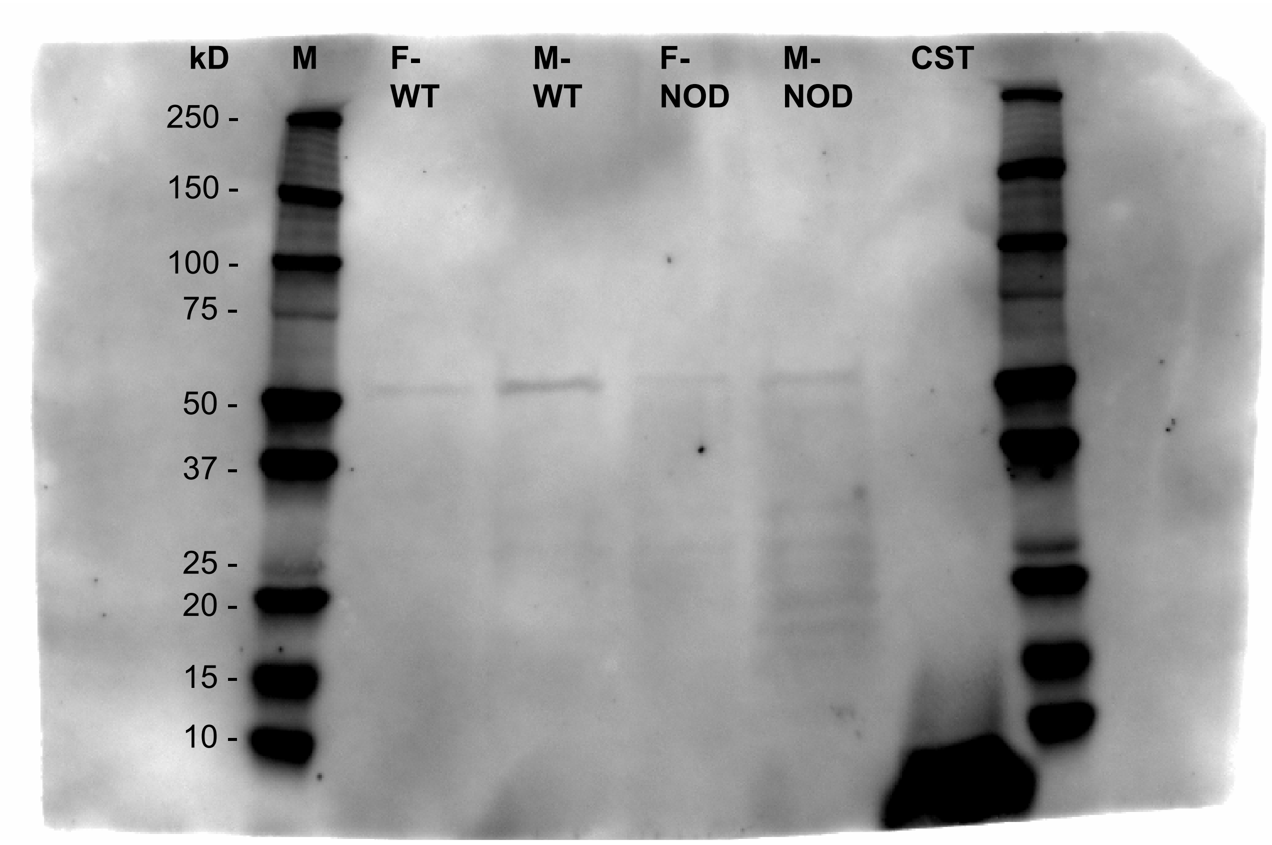

CST staining

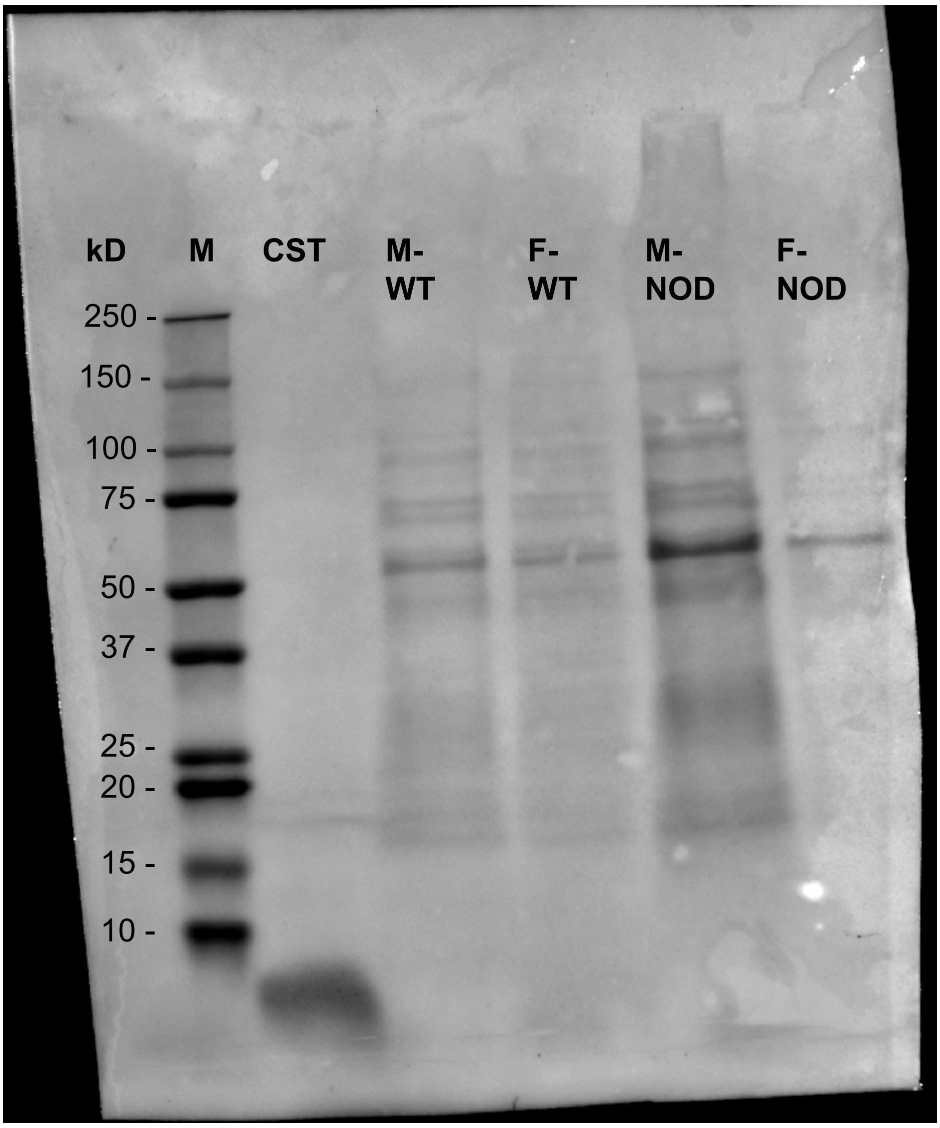

Protein staining

Supplementary Figure 1 Original immunoblot NOD mice from main Figure 1E. Original Western blot images showing catestatin staining or protein staining in wildtype (WT) or 8 week NOD mice (NOD) of Female (F) and Male (M) pancreas samples, including CST peptide (as positive control), Marker (M) and kilodalton (kD) values. n= 2 mice per group
.

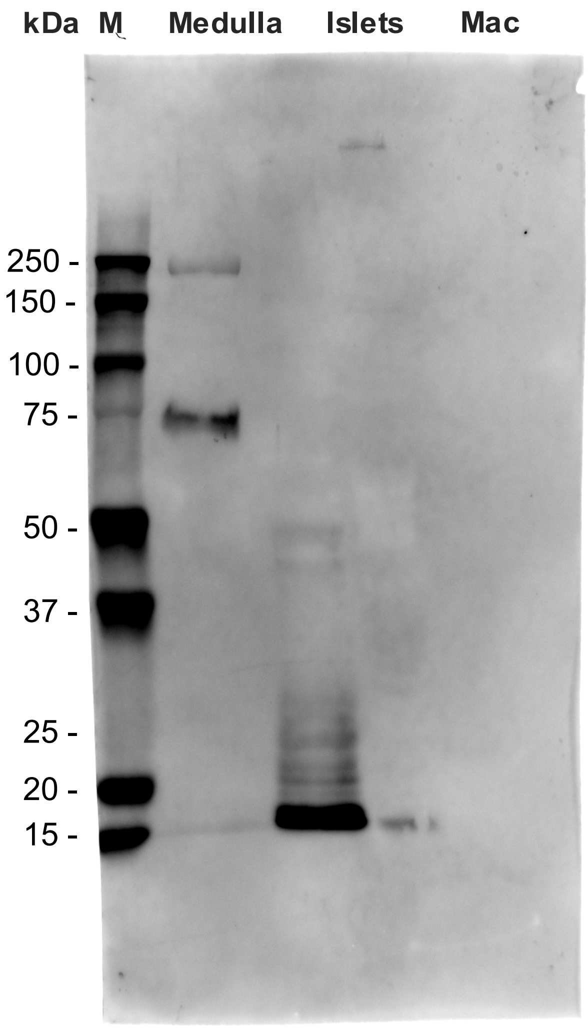

CgA staining

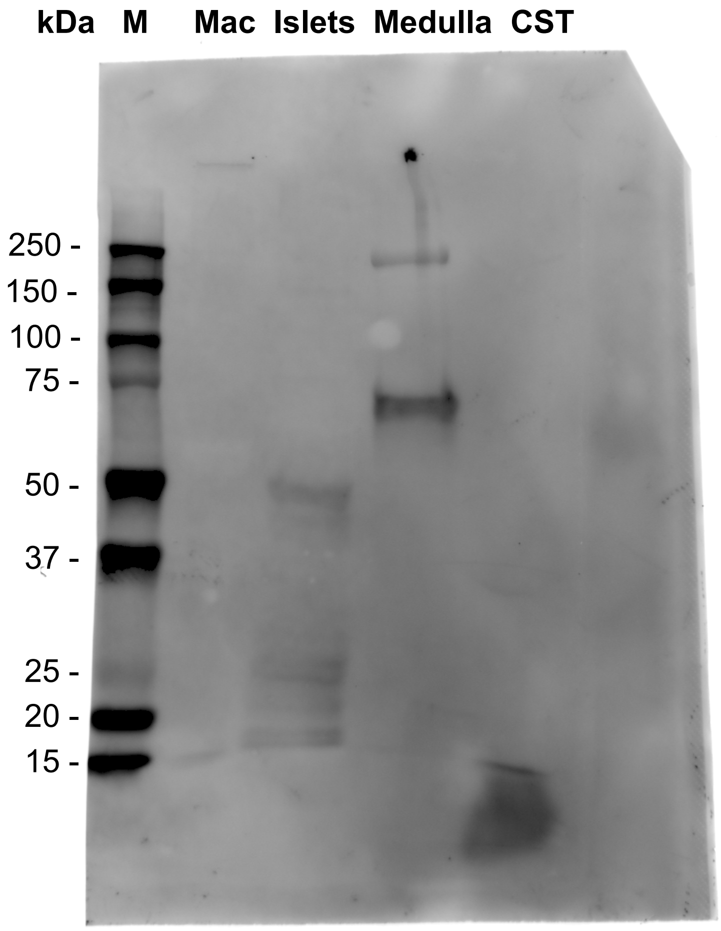

CST staining

Supplementary Figure 2 Original immunoblots from main Fig. 1F & G
Original Western blot images showing chromogranin A or catestatin staining in the adrenal medulla, pancreatic islets (Islets) and macrophages (mac), including CST peptide (as positive control), Marker (M) and kilodalton (kD) values. n=1 mouse per group

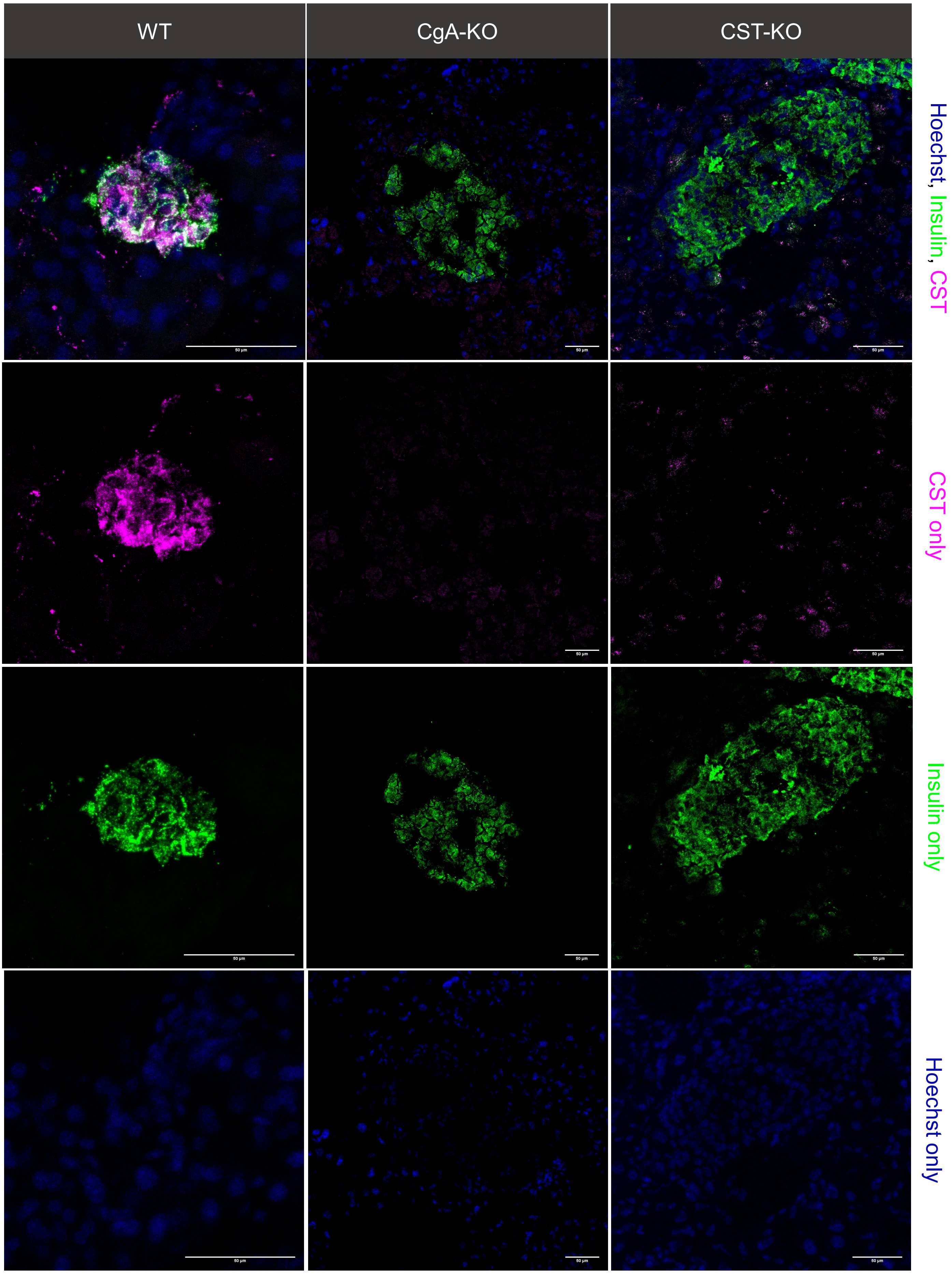

Supplementary Figure 3 Pancreatic islet CST staining confirming KO. Immunofluorescent staining for catestatin (pink), insulin (green), hoechst (blue) in WT, CgA-KO and CST-KO pancreas.

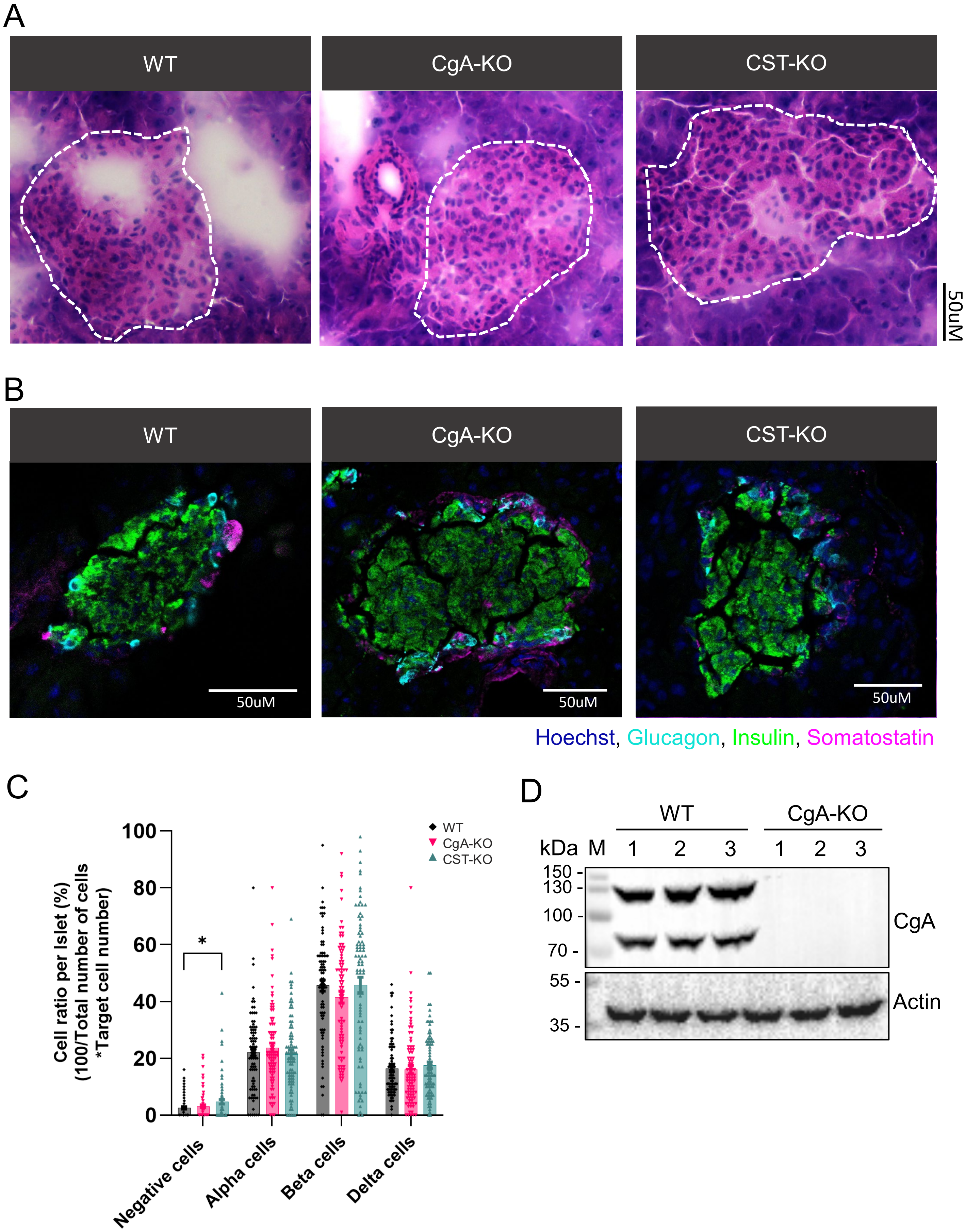

Supplementary Figure 4 Female pancreatic islet composition

A) H&E staining of female WT, CgA-KO and CST-KO pancreatic slices including annotations for islets (white dotted lines). **B)** Representative images of immunofluorescent staining of glucagon (cyan), insulin (green), somatostatin (magenta) and Hoechst (blue) on WT, CgA-KO and CST-KO pancreatic slices. **C)** Quantification of alpha/beta/delta/negative cells per islet. Quantification is based on the staining displayed in panel B. n=3 mice per group. **D)** Immunoblotting images showing CgA and actin staining in WT, CgA-KO and CST-KO mice including marker (M) and kilodalton (kDa) values. *P<0.05

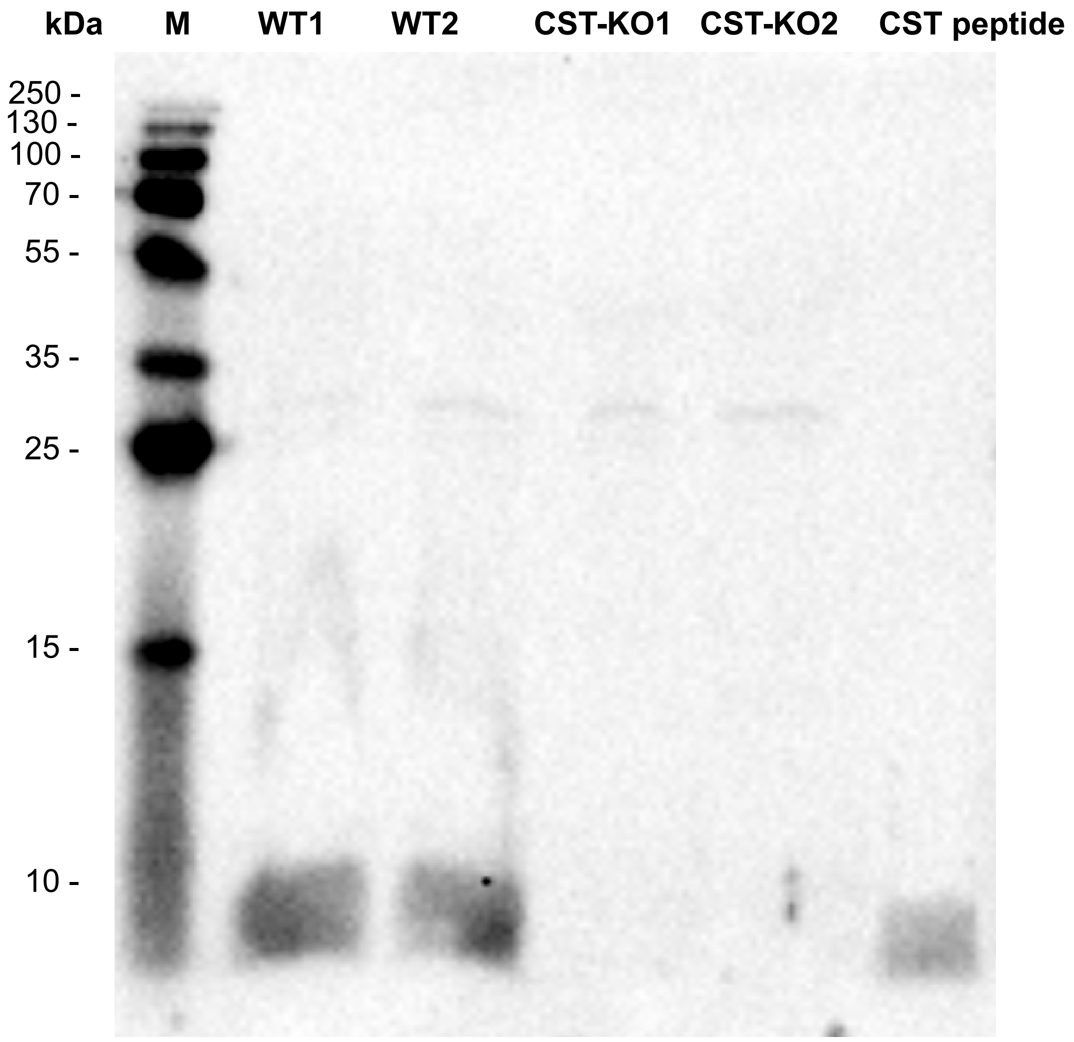

Catestatin staining

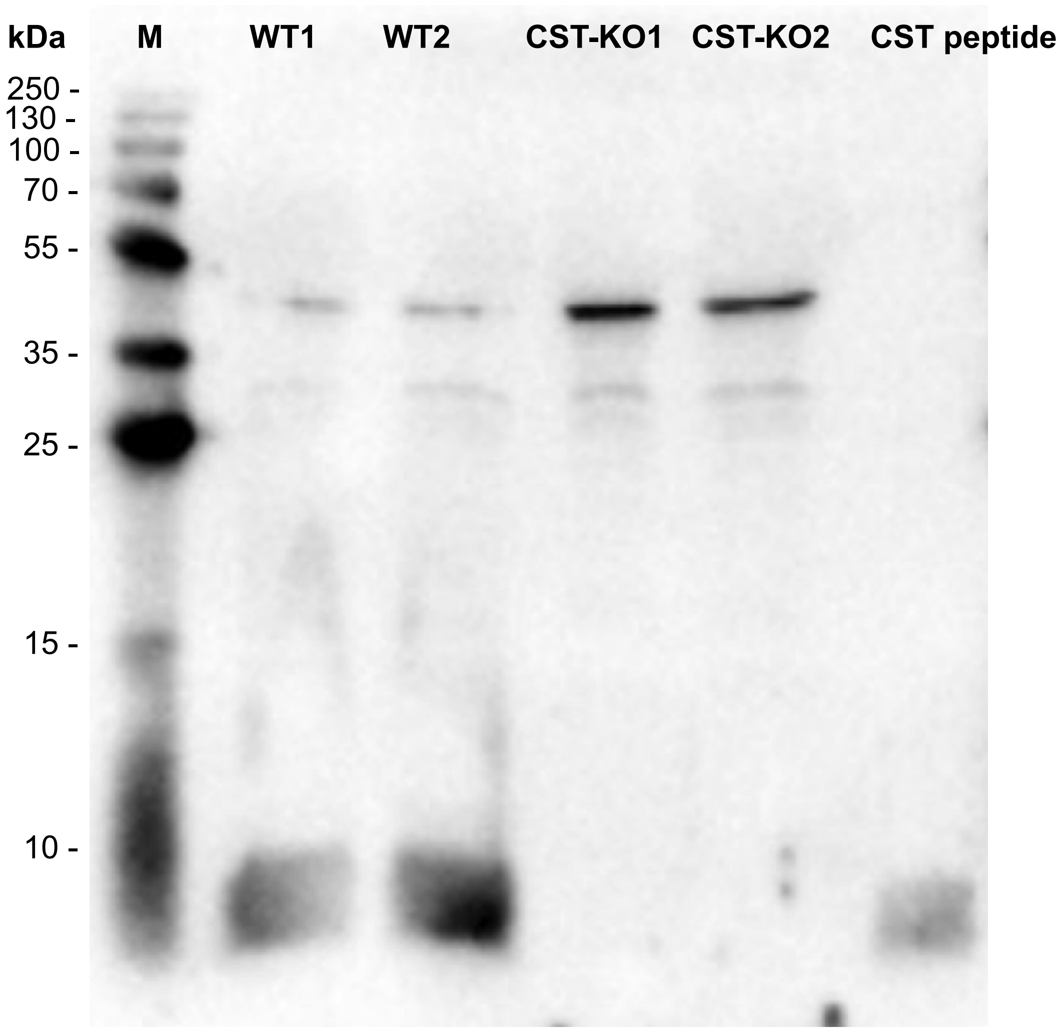

Actin staining

Supplementary Figure 5 Original immunoblots from main Fig. 2
Original immunoblotting images showing catestatin (CST) or actin staining for WT mice, CST-KO mice or CST peptide only (positive control) including marker (M) and kilodalton values (kDa). n= 2 mice pancreata per group

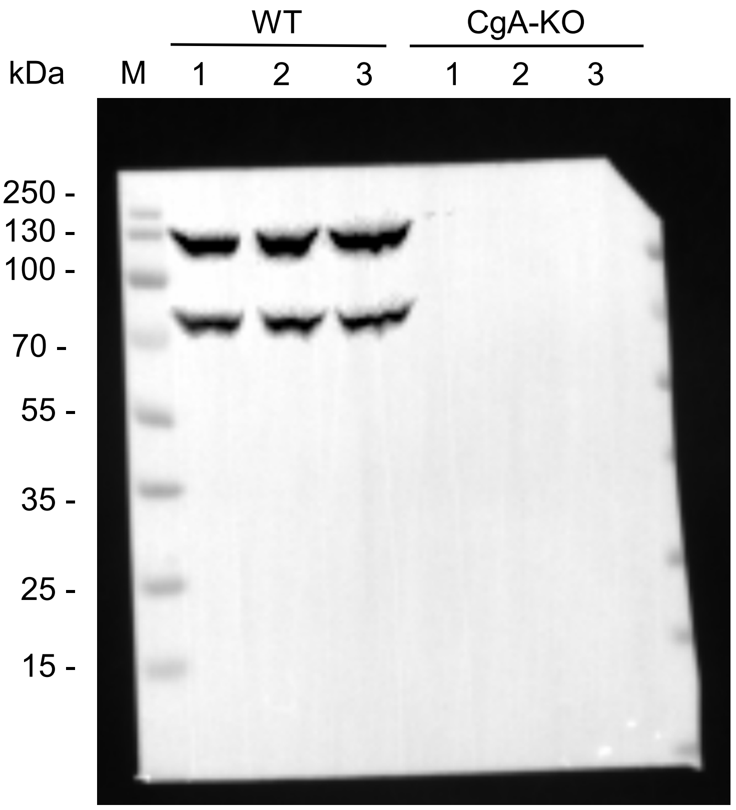

CgA staining

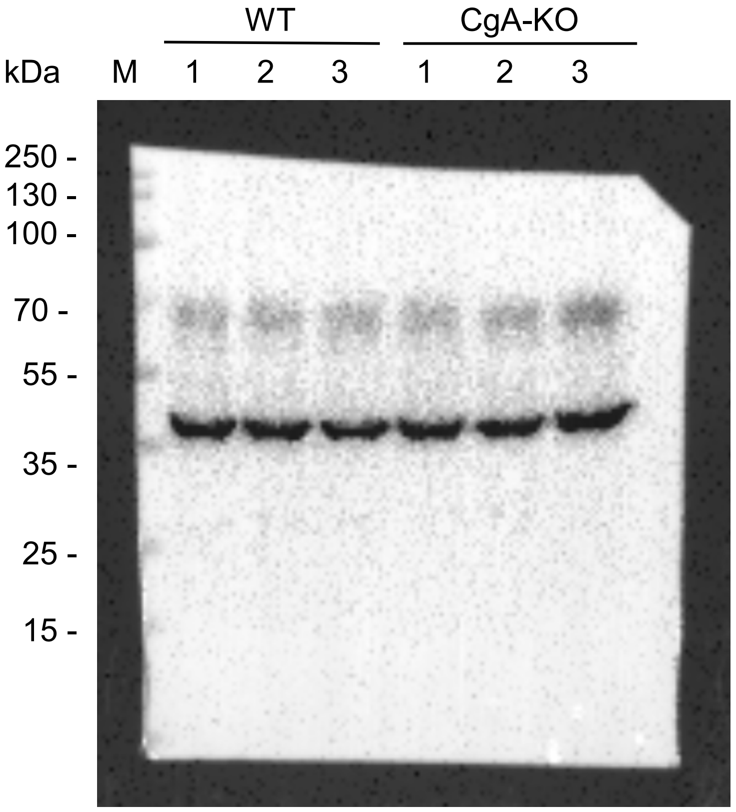

Actin staining

Supplementary Figure 6 Original immunoblots from Supplementary Figure 4 panel D

Original immunoblotting images showing chromogranin A or actin staining in WT and CgA-KO mice including marker (M) and kilodalton (kDa) values. n=3 mice pancreata per group

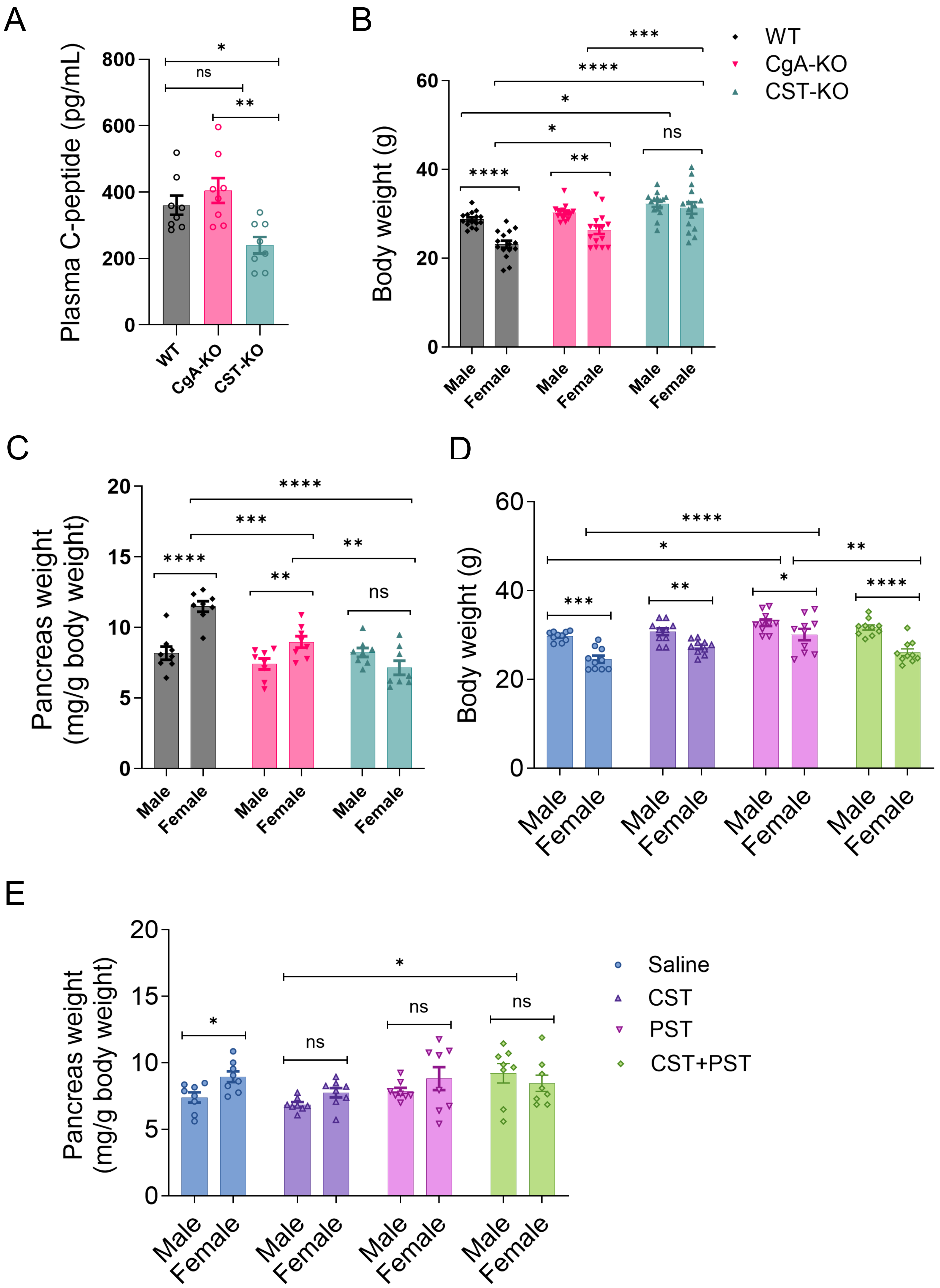

Supplementary Figure 7 Mice characteristics: peptide levels, pancreatic weight and body weight

A).C-peptide plasma levels (pg/mL). **B)** Male and female body weight (g) for WT, CgA-KO or CST-KO mice. N=15 **C))** Male and female pancreatic weight (mg/g body weight) for WT, CgA-KO or CST-KO mice. N=15 **D)** Body or pancreatic **E)** weight of CgA-KO male and female mice supplemented with saline (blue), CST (purple), PST (pink) or CST+PST (green). N=10. *p<0.05 , **p<0.01, ***p<0.001, ****p<0.0001

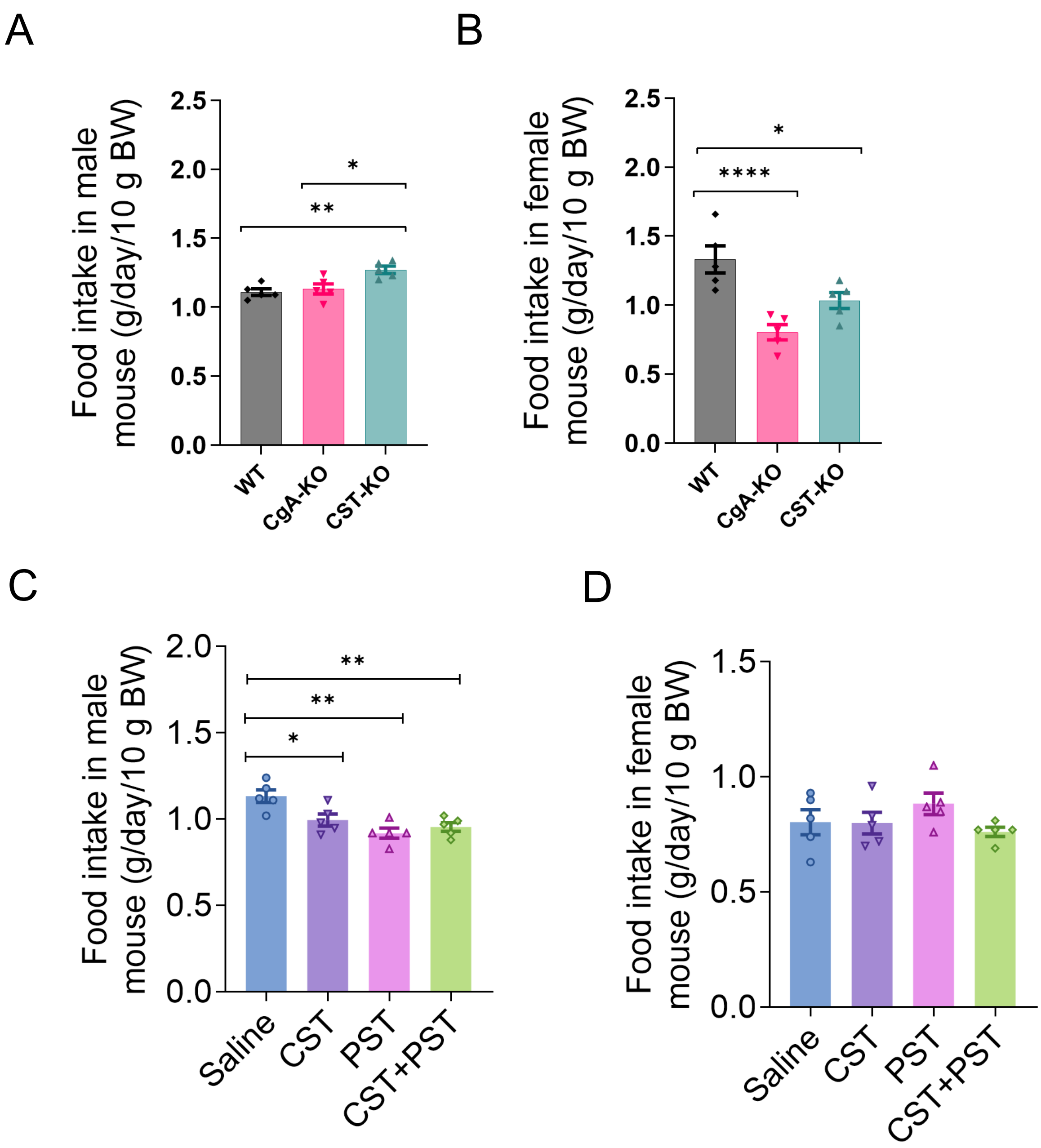

Supplementary Figure 8 Mice characteristics: food intake
A-B).Male and female food intake (g/day/10 g BW) measured for WT, CgA-KO or CST-KO mice. N=5 **C-D))** Male and female food intake (g/day/10 g BW) measured for CgA-KO male and female mice supplemented with saline, CST, PST or CST+PST. N=5. *p<0.05 , **p<0.01, ***p<0.001

A

Time (min)	WT vs CgA-KO	WT vs CST-KO	CgA-KO vs CST-KO
0	ns	ns	ns
15	*	**	****
30	***	**	****
60	**	**	****
90	ns	*	***
120	ns	ns	**

B

Time (min)	WT vs CgA-KO	WT vs CST-KO	CgA-KO vs CST-KO
0	ns	ns	ns
15	ns	**	****
30	**	***	****
60	***	***	****
90	**	***	****
120	*	**	****

C

Significance of CgA-KO supplemented with						
Time (min)	Sal vs CST	Sal vs PST	Sal vs CST+PST	CST vs PST	CST vs CST+PST	PST vs CST+PST
0	ns	ns	ns	ns	ns	ns
15	ns	**	ns	****	ns	**
30	ns	****	ns	****	*	****
60	ns	****	ns	****	ns	****
90	ns	****	ns	****	ns	****
120	ns	***	ns	****	ns	***

Supplementary Figure 9 Significance Male GTT and PTT data from main Fig. 3
A) GTT significance table from GTT graph panel A comparing blood glucose levels (mg/dL) over time (min) of WT, CgA-KO and CST-KO male mice. **B)** Pyruvate tolerance test (PTT) significance table from PTT graph panel C comparing blood glucose levels of WT, CgA-KO and CST-KO male mice. **C)** PTT significance table from PTT graph showing blood glucose results (mg/dL) over time (min) of male CgA-KO mice supplemented with saline, CST, PST or CST+PST N=10 per group. *$p<0.05$, **$p<0.01$, ***$p<0.001$, ****$p<0.0001$

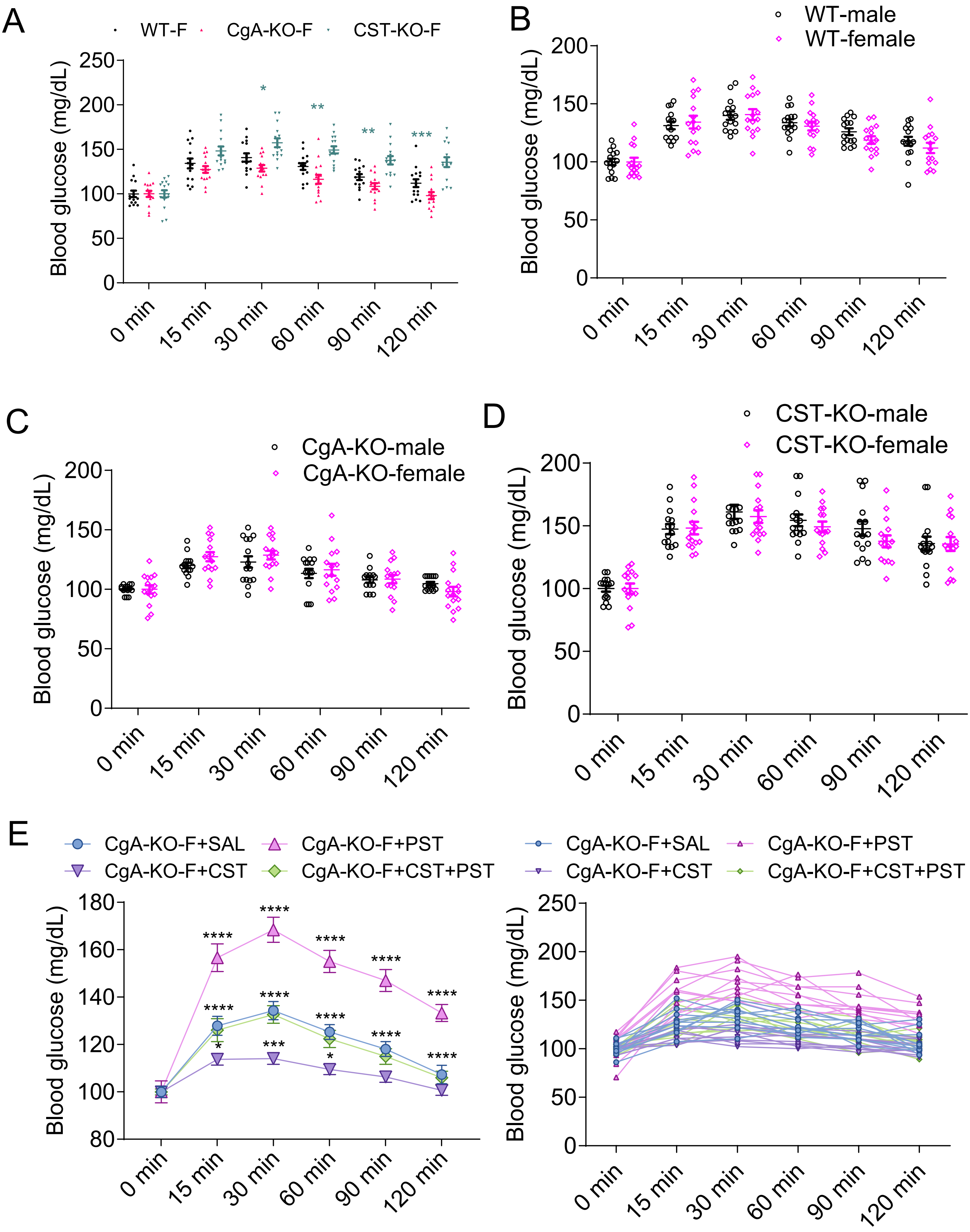

Supplementary Figure 10 Female PTT data and male and female comparison
A) Graph displaying pyruvate tolerance test (PTT) blood glucose results (mg/dL) for female (F) WT (black), CgA-KO (pink) and CST-KO (green) over time (min) N=15 per group. **B-D)** Graphs displaying combined data of PTT, comparing males and females WT, CgA-KO and CST-KO blood glucose results (mg/dL) over time (min). **E)** Graph displaying combined or individual data of PTT blood glucose results (mg/dL) over time (min) of female (F) CgA-KO mice supplemented with saline (blue), CST (purple), PST (pink) or CST+PST (green) N=10 per group. $^*p<0.05$, $^{**}p<0.01$, $^{***}p<0.001$, $^{****}p<0.0001$

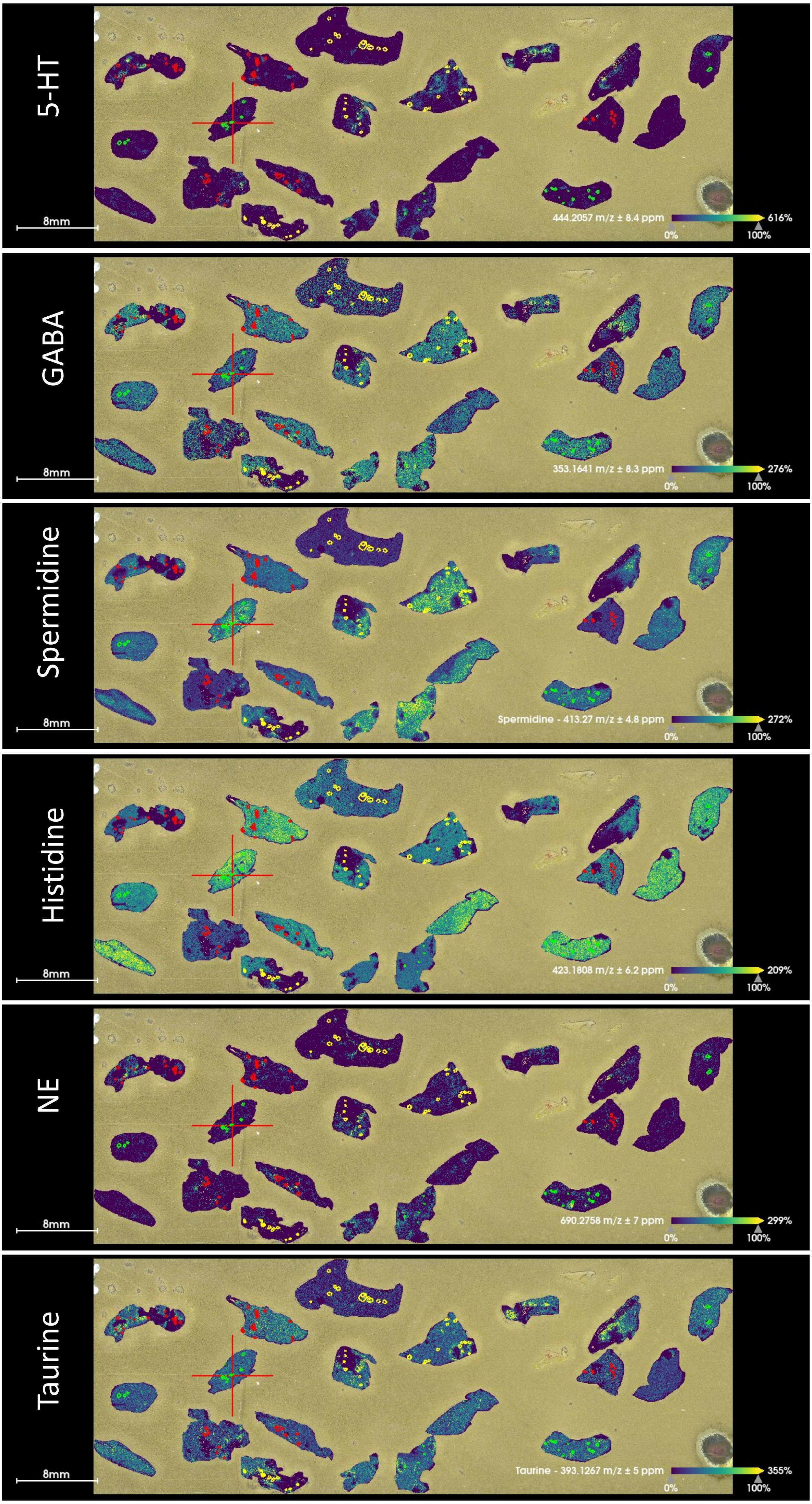

Supplementary Figure 11 Spatial MS heatmaps of analytes identified in the pancreas

Spatial MS full heatmaps showing all measured pancreata including annotated islet and exocrine areas for WT (red), CgA-KO (green), CST-KO (yellow).

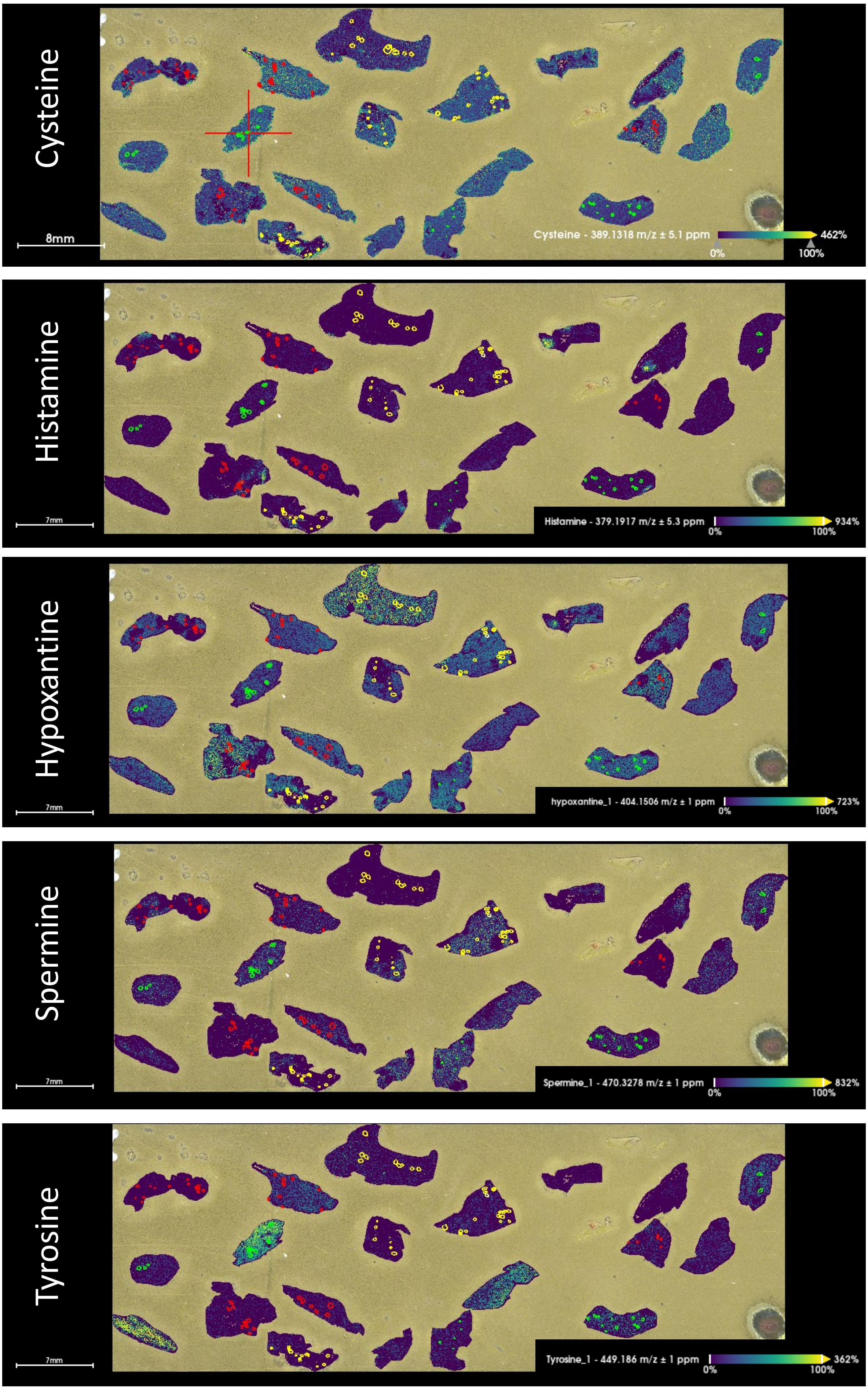

Supplementary Figure 12 Spatial MS heatmaps of analytes identified in the pancreas
Spatial MS full heatmaps showing all measured pancreata including annotated islet and exocrine areas for WT (red), CgA-KO (green), CST-KO (yellow).

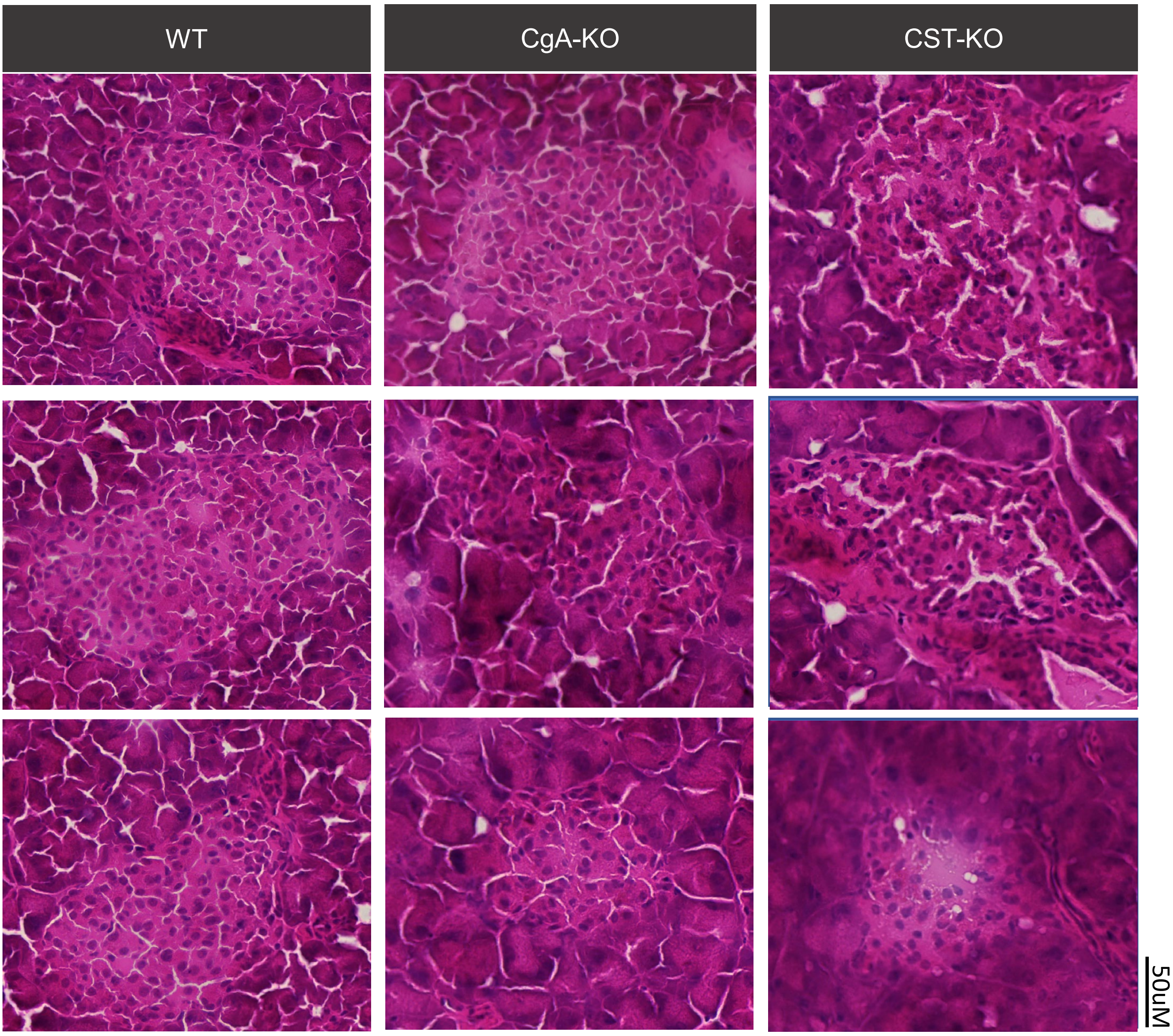

Supplementary Figure 13 H&E stain for spatial MS of male pancreatic islets. Consecutive sections from these were used for mass spectrometry imaging and the H&E stainings here guided islet annotations (see Supplementary Method Fig 2).

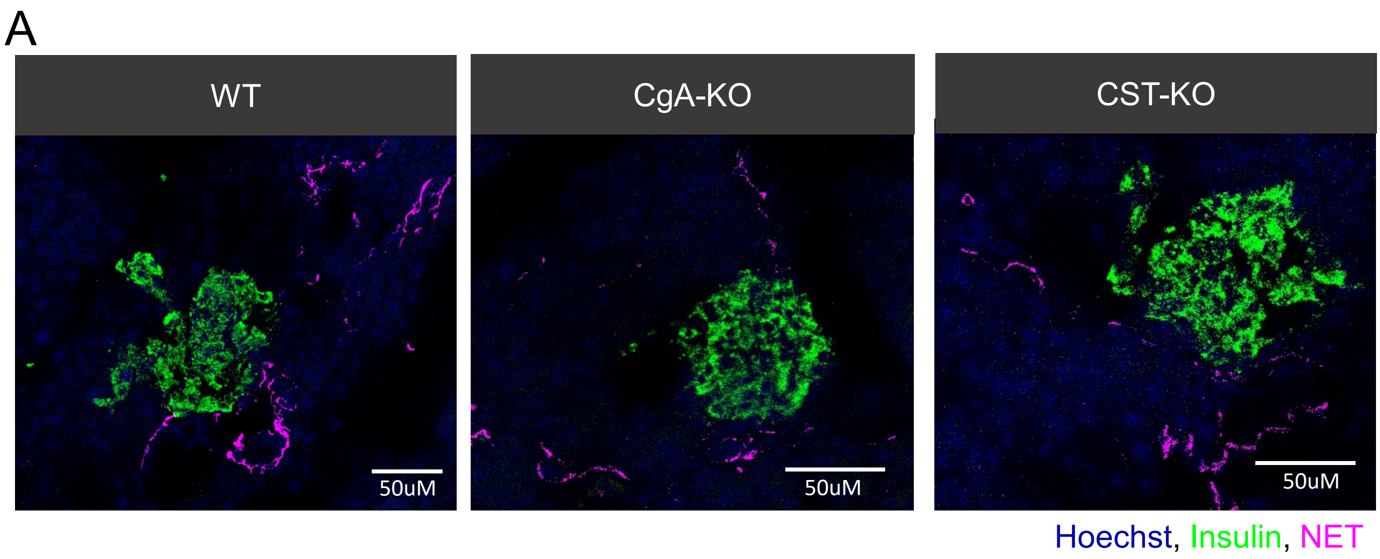

Supplementary Figure 14 Female innervation of pancreatic islets
Representative images of nerve staining (norepinephrine transporter (NET, magenta), islets (insulin, green) and nuclei (Hoechst) in WT, CgA-KO and CST-KO pancreatic slices.

Annotation of islets based on staining in all channels

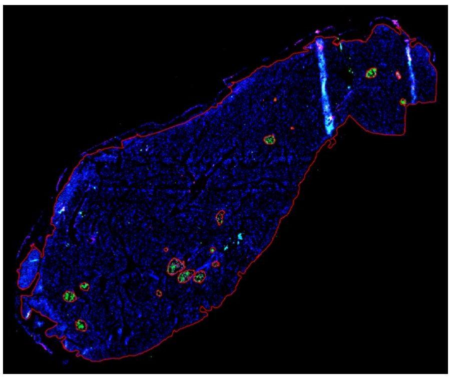

Perform cell detection on annotated islets by training classifier

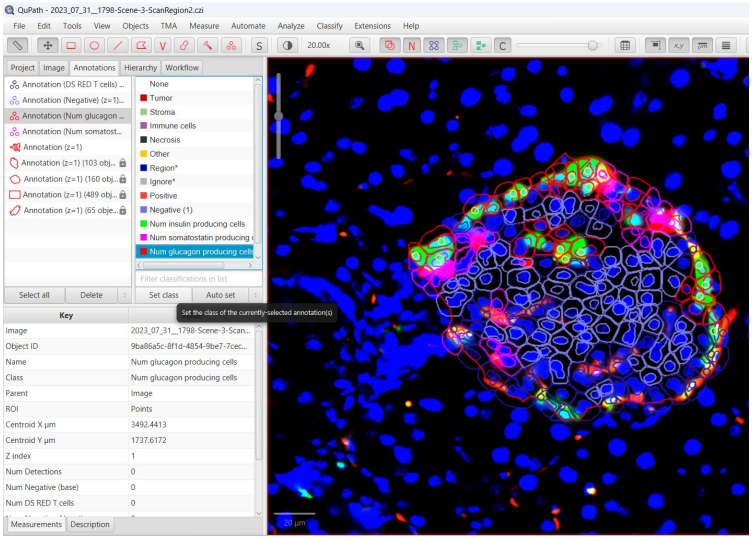

Settings for cell detection

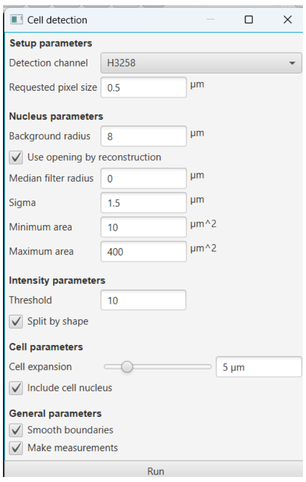

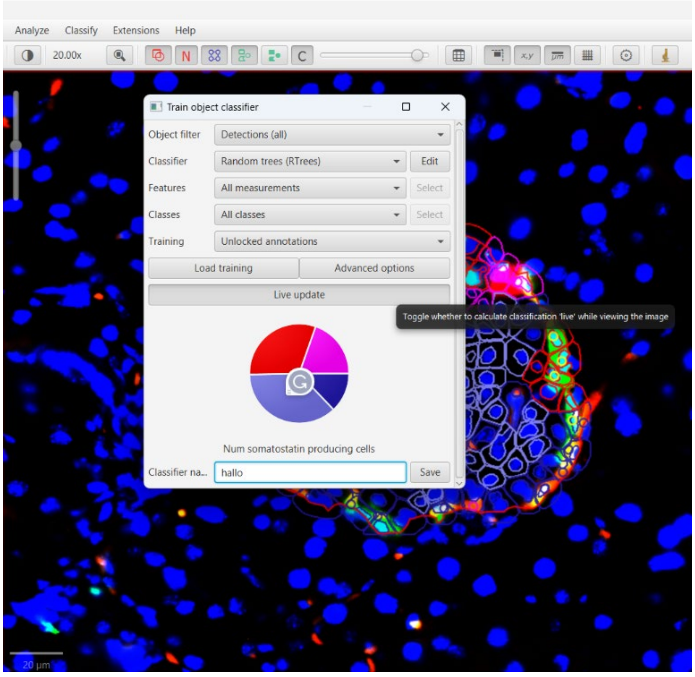

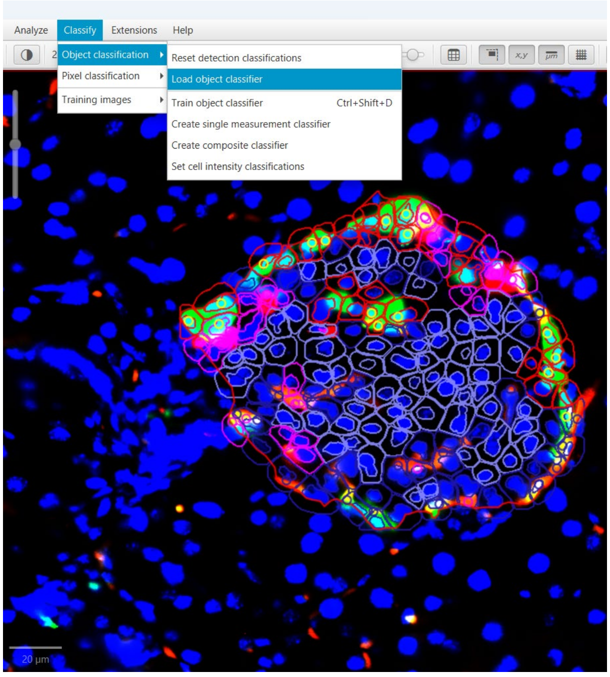

Example of classified islet

Obtained cell detection using classifier

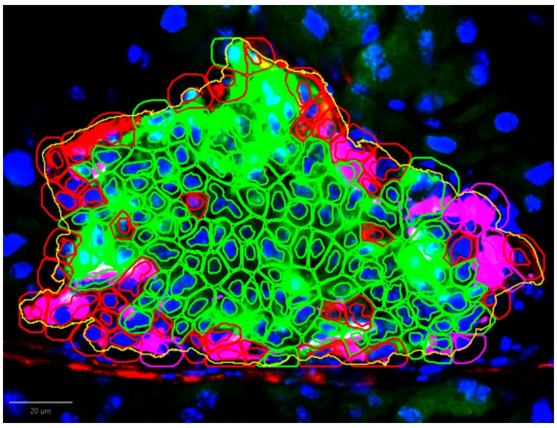

Legend
Insulin
Glucagon
Somatostatin
Negative

Original fluorescent staining

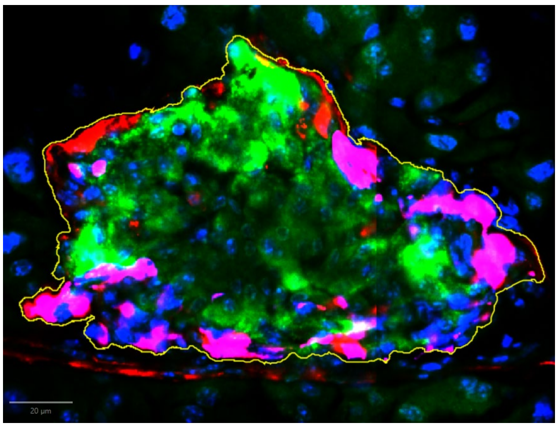

Supplementary Method Figure 1 QuPath workflow for alpha/beta/delta cell detection
Step-by-step workflow for cell detection, annotation and quantification of alpha/beta/delta cells in the pancreatic islet using QuPath.

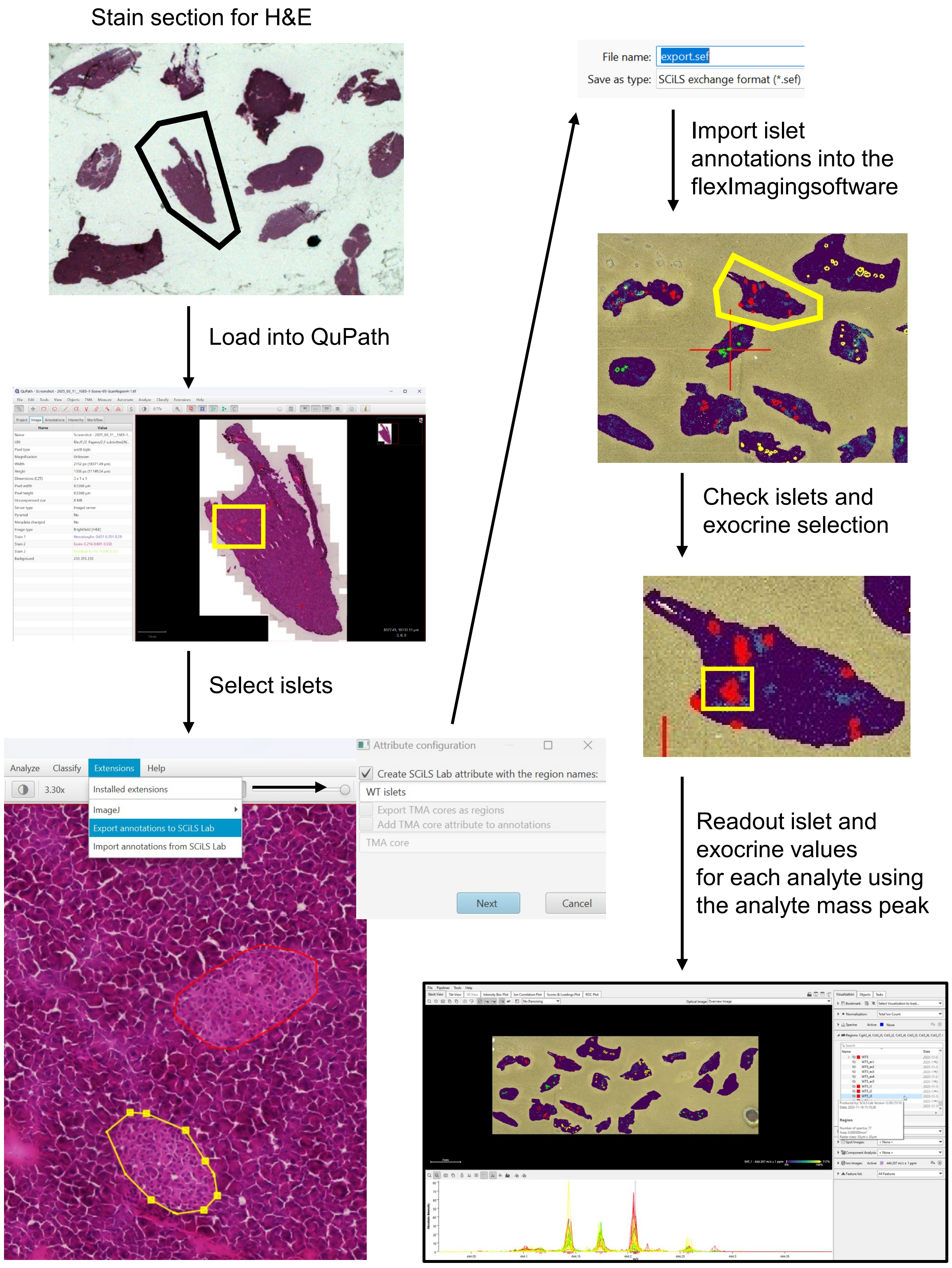

Supplementary Method Figure 2 Spatial MS workflow
Step-by-step workflow for islet annotation in spatial MS data using both H&E stained consecutive sections and serotonin quantity.

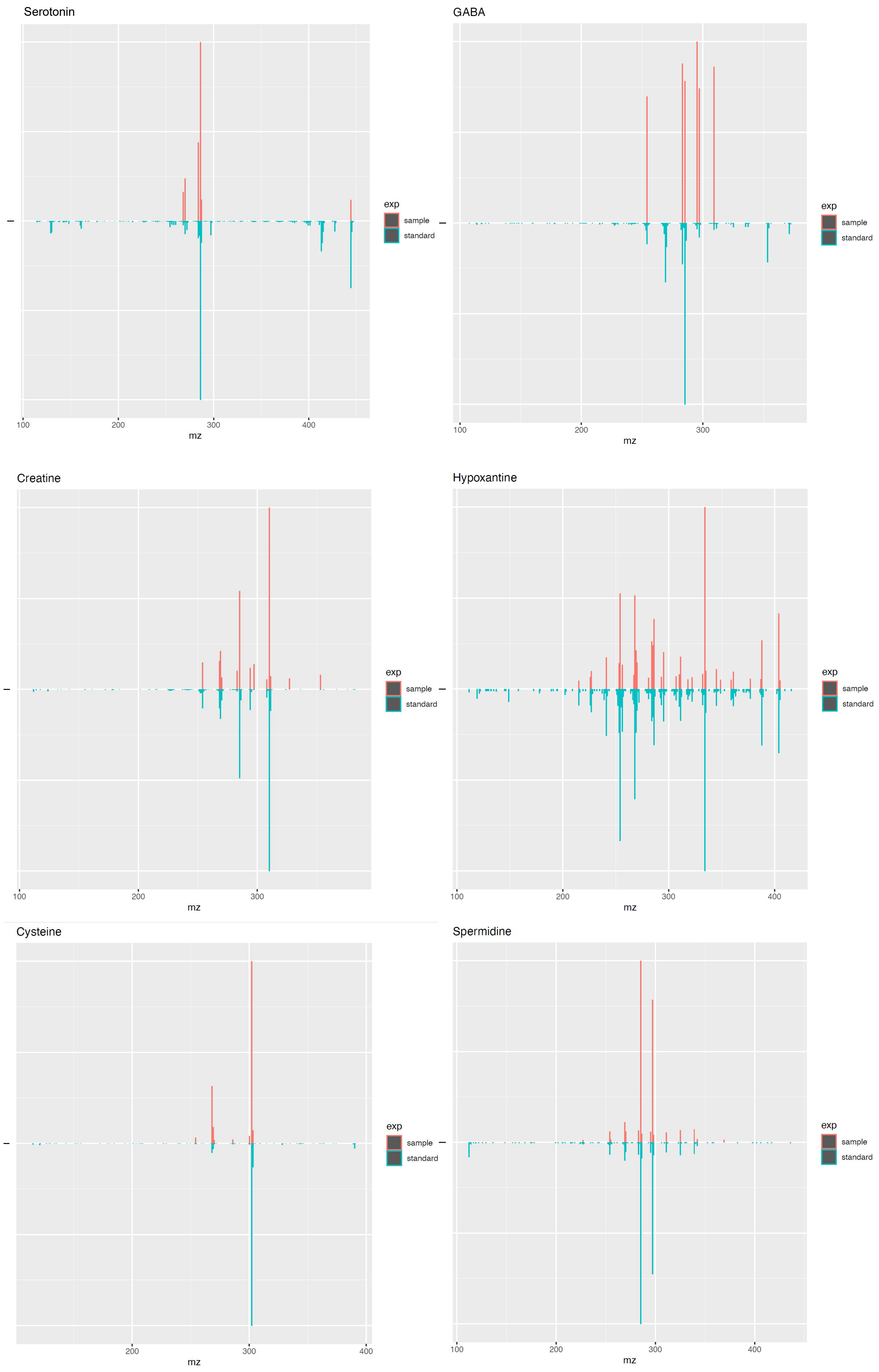

Supplementary Method Figure 3 MS/MS plots of analytes identified in the pancreas Red lines show pancreas sample signal and blue lines show signal of the corresponding control standard.

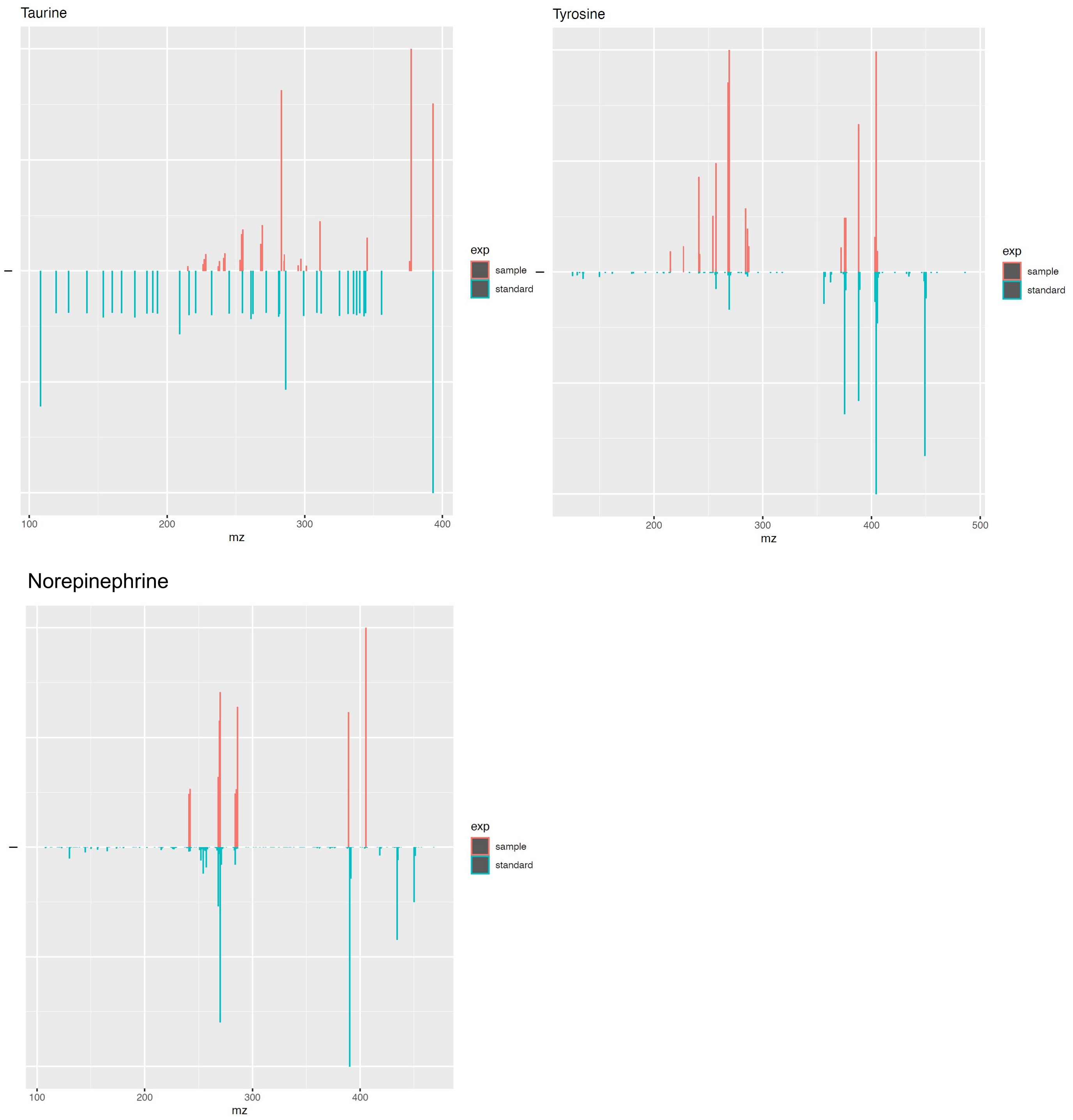

Supplementary Method Figure 4 MS/MS plots of analytes identified in the pancreas 2
Red lines show pancreas sample signal and blue lines show signal of the corresponding control standard.
