# Supplementary material for: Chromogranin A and catestatin regulate pancreatic islet homeostasis, endocrine function, and neurotransmitter signaling
Source: Commun Biol. 2025 Nov 26;8:1684. doi: 10.1038/s42003-025-09135-z (PMC12658243; doi:10.1038/s42003-025-09135-z)
Supplement: Supplementary file 2 — Reporting summary [file 42003_2025_9135_MOESM2_ESM.pdf]

nature portfolio

Corresponding author(s): Gustaf Christoffersson
Sushil Mahata

Last updated by author(s): Sep 29, 2025

Reporting Summary

Nature Portfolio wishes to improve the reproducibility of the work that we publish. This form provides structure for consistency and transparency in reporting. For further information on Nature Portfolio policies, see our Editorial Policies and the Editorial Policy Checklist.

Statistics

For all statistical analyses, confirm that the following items are present in the figure legend, table legend, main text, or Methods section.

n/a	Confirmed	
☐	☒	The exact sample size (*n*) for each experimental group/condition, given as a discrete number and unit of measurement
☐	☒	A statement on whether measurements were taken from distinct samples or whether the same sample was measured repeatedly
☐	☒	The statistical test(s) used AND whether they are one- or two-sided<br>*Only common tests should be described solely by name; describe more complex techniques in the Methods section.*
☒	☐	A description of all covariates tested
☐	☒	A description of any assumptions or corrections, such as tests of normality and adjustment for multiple comparisons
☐	☒	A full description of the statistical parameters including central tendency (e.g. means) or other basic estimates (e.g. regression coefficient) AND variation (e.g. standard deviation) or associated estimates of uncertainty (e.g. confidence intervals)
☐	☒	For null hypothesis testing, the test statistic (e.g. *F*, *t*, *r*) with confidence intervals, effect sizes, degrees of freedom and *P* value noted<br>*Give P values as exact values whenever suitable.*
☒	☐	For Bayesian analysis, information on the choice of priors and Markov chain Monte Carlo settings
☒	☐	For hierarchical and complex designs, identification of the appropriate level for tests and full reporting of outcomes
☒	☐	Estimates of effect sizes (e.g. Cohen's *d*, Pearson's *r*), indicating how they were calculated

Our web collection on statistics for biologists contains articles on many of the points above.

Software and code

Policy information about availability of computer code

Data collection	Annotation from image analysis software QuPath (open source) was integrated into Bruker's SCiLS mass spectrometry imaging software through a built-in plugin.
Data analysis	Image analysis was performed using Imaris 9.7 and QuPath. Statistical analysis was performed using GraphPad Prism 10.

For manuscripts utilizing custom algorithms or software that are central to the research but not yet described in published literature, software must be made available to editors and reviewers. We strongly encourage code deposition in a community repository (e.g. GitHub). See the Nature Portfolio guidelines for submitting code & software for further information.

Data

Policy information about availability of data

All manuscripts must include a data availability statement. This statement should provide the following information, where applicable:

- Accession codes, unique identifiers, or web links for publicly available datasets
- A description of any restrictions on data availability
- For clinical datasets or third party data, please ensure that the statement adheres to our policy

Data will be publicly available through FigShare: https://figshare.com/s/8dace17885070d6916f1. Raw spatial MS data, slide scans and human data are available upon request via Gustaf Christoffersson (gustaf.christoffersson@scilifelab.uu.se).

Research involving human participants, their data, or biological material

Policy information about studies with human participants or human data. See also policy information about sex, gender (identity/presentation), and sexual orientation and race, ethnicity and racism.

Reporting on sex and gender	Blood plasma of male and female participants was collected with informed consent.
Reporting on race, ethnicity, or other socially relevant groupings	This information was not collected on the participants.
Population characteristics	Participants were age- and sex-matched. All human participant characteristics are available in Supplementary Table 1.
Recruitment	Blood plasma of healthy and diabetes type 1 participants was collected with informed consent and stored at a biobank.
Ethics oversight	Uppsala Regional Ethical Review Board

Note that full information on the approval of the study protocol must also be provided in the manuscript.

Field-specific reporting

Please select the one below that is the best fit for your research. If you are not sure, read the appropriate sections before making your selection.

☒ Life sciences ☐ Behavioural & social sciences ☐ Ecological, evolutionary & environmental sciences

For a reference copy of the document with all sections, see nature.com/documents/nr-reporting-summary-flat.pdf

Life sciences study design

All studies must disclose on these points even when the disclosure is negative.

Sample size	Sample sizes were determined through power calculations with type I/II error rates of alpha 0.05 and beta 0.2.
Data exclusions	No data exclusions
Replication	All experiments and analyses were either repeated at least twice, or contained at least two technical replicates.
Randomization	n/a
Blinding	Genotypes were blinded to both experimenters performing analyses and people performing quantifications.

Reporting for specific materials, systems and methods

We require information from authors about some types of materials, experimental systems and methods used in many studies. Here, indicate whether each material, system or method listed is relevant to your study. If you are not sure if a list item applies to your research, read the appropriate section before selecting a response.

Materials & experimental systems

n/a	Involved in the study	
☐	☒	Antibodies
☒	☐	Eukaryotic cell lines
☒	☐	Palaeontology and archaeology
☐	☒	Animals and other organisms
☒	☐	Clinical data
☒	☐	Dual use research of concern
☒	☐	Plants

Methods

n/a	Involved in the study	
☒	☐	ChIP-seq
☒	☐	Flow cytometry
☒	☐	MRI-based neuroimaging

Antibodies

Antibodies used	guinea pig insulin primary antibody Fitzgerald; 20-IP35, mouse anti-glucagon Proteintech; 67286-1-Ig, rabbit anti-somatostatin, Abcam; ab111912, rabbit anti-norephrine transporter Abcam; ab254361, rat anti-IBA1 Synaptic Systems; 234017, rabbit anti-Tyrosine Hydroxylase Abcam; ab137869, 488-anti-mouse CD3, Biolegend; 100210, rabbit anti-CST, Proteintech; 289-MM-0288, U-

	PLEX antibodies K152ACM-1: Mouse/Rat C-peptide, Glucagon, Mouse IL-1beta, Mouse IL-6, Mouse IL-10, Mouse/Rat Insulin, Mouse KC/GRO, Mouse/Rat Leptin, MCP-1, TNF-alpha
Validation	All antibodies were used in accordance with methods validated by the manufacturers. Antibodies were further validated and titrated in lab for their respective use.

Animals and other research organisms

Policy information about studies involving animals; ARRIVE guidelines recommended for reporting animal research, and Sex and Gender in Research

Laboratory animals	Mice
Wild animals	n/a
Reporting on sex	Male and female mice were used in this study.
Field-collected samples	n/a
Ethics oversight	All animal experiments were approved by the Institutional Animal Care and Use Committee at UCSD, and the regional animal ethics committee in Uppsala, Sweden.

Note that full information on the approval of the study protocol must also be provided in the manuscript.

Plants

Seed stocks	*Report on the source of all seed stocks or other plant material used. If applicable, state the seed stock centre and catalogue number. If plant specimens were collected from the field, describe the collection location, date and sampling procedures.*
Novel plant genotypes	*Describe the methods by which all novel plant genotypes were produced. This includes those generated by transgenic approaches, gene editing, chemical/radiation-based mutagenesis and hybridization. For transgenic lines, describe the transformation method, the number of independent lines analyzed and the generation upon which experiments were performed. For gene-edited lines, describe the editor used, the endogenous sequence targeted for editing, the targeting guide RNA sequence (if applicable) and how the editor was applied.*
Authentication	*Describe any authentication procedures for each seed stock used or novel genotype generated. Describe any experiments used to assess the effect of a mutation and, where applicable, how potential secondary effects (e.g. second site T-DNA insertions, mosiacism, off-target gene editing) were examined.*
